# Evaluation of time to antibiotic therapy after implementation of an updated rapid diagnostic test for gram-negative bacteremia

**DOI:** 10.1128/jcm.00328-26

**Published:** 2026-08-31

**Authors:** Rawan Hussein, Leslie Lee, Rajat Nog, Donald Chen, Vishnu Chaturvedi, Nicholas Feola

**Affiliations:** 1 Department of Clinical Pharmacy Services, Westchester Medical Center 8138, Valhalla, New York, USA; 2 Department of Infectious Diseases Services, Westchester Medical Center 8138, Valhalla, New York, USA; 3 Department of Pathology, Westchester Medical Center, Valhalla, New York, USA; Mayo Clinic, Rochester, Minnesota, USA

**Keywords:** rapid diagnostics, bacteremia, bloodstream infection, BCID 2, BCID

## Abstract

Rapid pathogen identification optimizes antibiotic therapy in bacteremia and can be achieved through molecular-based diagnostic tests. This study assesses the clinical impact after the implementation of an updated multiplex PCR panel (BIOFIRE Blood Culture Identification 2, BCID2). This single-center, retrospective, quasi-experimental study compared outcomes in adult patients with gram-negative bloodstream infections (GN-BSIs) pre and post BCID2 implementation. Data were collected from 1 March 2022 to 1 March 2024. Patients were included if they were ≥18 years of age, had a gram-negative organism identified by the panels, and received ≥48 h of antibiotics. The primary outcome was time to effective antimicrobial therapy; secondary outcomes included time to appropriate therapy, 30-day mortality, and hospital length of stay. A total of 180 patients were included (90 per BCID and BCID2 groups). Baseline characteristics were similar: median age 62 years, mostly non-critically ill, Charlson Comorbidity Index 4, and Pitt Bacteremia score 1 in both groups. Median time to effective therapy was reduced in the BCID2 vs BCID group (1 vs 4 h, *P* = 0.004) as well as time to appropriate therapy (25 vs 49 h, *P* = 0.627). In the ESBL subgroup, BCID2 significantly reduced time to effective therapy (0.5 vs 3 h, *P* = 0.006) and time to appropriate therapy (16 vs 44 h, *P* = 0.016). There was no significant difference in mortality (6.7% vs 8.9%, *P* = 0.756) or length of stay (10 vs 14 days, *P* = 0.134) between BCID and BCID2 groups. The implementation of BCID2 significantly reduced time to effective therapy in GN-BSIs, particularly among resistant organisms.

**IMPORTANCE:**

This study evaluated the impact of transitioning from BCID to BCID2 panel in gram-negative bloodstream infections to address gaps in resistance detection and assess whether expanded molecular diagnostics improve time to antibiotic therapy and clinical outcomes.

## INTRODUCTION

Prompt pathogen identification is essential for initiating effective therapy and improving patient outcomes in bacteremic patients. Delays in appropriate antimicrobial treatment are associated with increased mortality rates in patients with blood stream infections (BSIs) (1, 2). Conventional pathogen identification methods and susceptibility testing rely on culture-based techniques which may require more than 72 h to yield final results. During this time, patients initiated on broad-spectrum empiric antimicrobials may be at risk for receiving suboptimal therapy and potential increased risk of developing antimicrobial resistance (3–6).

Molecular rapid diagnostic tests (mRDTs) have emerged as important tools improving the management of infectious diseases by addressing the limitations of conventional methods. Nucleic acid amplification techniques, such as polymerase chain reaction (PCR), are utilized to detect bacterial or fungal DNA directly from positive blood culture bottles. These assays are able to identify pathogens and select resistance markers within hours of bacterial growth, rather than days, when compared to conventional methods. Multiple studies have demonstrated that mRDTs, when implemented alongside antimicrobial stewardship review, significantly reduce time to effective therapy and improve patient outcomes (4, 7, 8). Current guidelines recommend that clinical laboratories implement rapid diagnostics in combination with a comprehensive plan to actively communicate the actionable test results in order to decrease time to targeted therapy and length of stay (9).

The BioFire Blood Culture Identification (BCID) panel (BioFire Diagnostics, Salt Lake City, UT, USA) is a multiplex polymerase chain reaction (PCR) assay designed to rapidly identify pathogens and detect resistance markers directly from positive blood cultures. The BCID panel tests for a comprehensive list of 24 pathogens and 3 antibiotic resistance genes (mecA, vanA/B, and KPC). Studies on BCID panel demonstrated reduced time to effective and appropriate therapy compared to conventional identification methods, particularly when used in conjunction with Antimicrobial Stewardship Programs (ASPs) (10). The BCID panel has less comprehensive detection for extended-spectrum beta-lactamase (ESBL) and carbapenemase genes, which may limit its use in healthcare settings with a high prevalence of multidrug-resistant organisms. The BCID2 panel was developed to address these limitations, expanding detection to 43 targets total—33 pathogens and 10 resistance markers (11). The additional resistance markers include IMP, OXA-48, NDM, VIM, mcr-1, and CTX-M (12). Rapid identification of these genes may facilitate earlier definitive therapy and improved outcomes.

Studies evaluating the impact of transitioning from BCID to the newer BCID2 panel remain limited but promising (12, 13). Importantly, two simulation-based analyses assessed the theoretical clinical utility of BCID2, highlighting its potential to reduce time to definitive therapy, through rapid pathogen identification. While both studies offered additional insight into the expanded coverage of BCID2, their findings were derived from hypothetical decision trees, rather than real-world clinical outcomes. As a result, they were unable to assess downstream patient-centered outcomes such as mortality, or hospital length of stay (14, 15).

This study aims to provide real-world evidence comparing the original BCID panel to the BCID2 panel in patients with gram-negative bloodstream infections. By evaluating both microbiologic and clinical endpoints, this study seeks to assess the true clinical impact of BCID2 implementation in a tertiary care setting.

## MATERIALS AND METHODS

This was an institutional review board (IRB) approved retrospective, quasi-experimental study conducted at Westchester Medical Center, a 654-bed tertiary and quaternary academic medical center, in a suburb of New York City. The study aimed to evaluate the clinical impact of implementing the BCID2 panel in patients with gram-negative bloodstream infections (GN-BSIs). Included patients were adults (≥18 years) with at least one positive blood culture with a gram-negative organism identified via the BCID or BCID2 panel, first positive blood culture during hospital admission, and received antibiotic therapy for at least 48 h for treatment of this bloodstream infection. Patients were excluded if the positive blood cultures were deemed contaminants, infection was polymicrobial, non-gram-negative organisms, antibiotics were initiated prior to blood culture collection, cultures obtained at an outside facility, or if the patient expired or was discharged prior to organism identification.

The pre-implementation period extended from 1 March 2022 to 1 March 2023, including patients whose blood cultures underwent pathogen identification via the original BCID panel. The post-implementation period extended from 2 March 2023 to 1 March 2024 and included patients whose blood cultures underwent pathogen identification via the BCID2 panel, which replaced the original BCID panel on 2 March 2023. Both panels were used in conjunction with conventional detection methods and antimicrobial stewardship review conducted during business hours from Monday through Friday.

**Fig 1:**
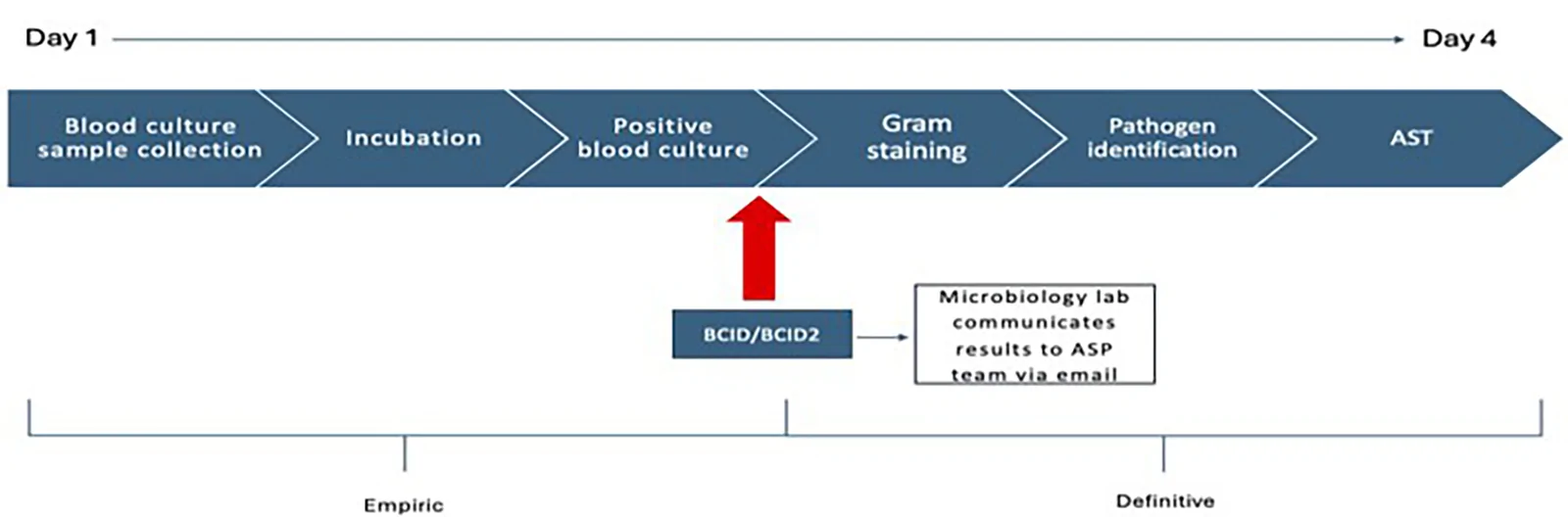
Blood culture identification workflow. Workflow illustrating the diagnostic and antimicrobial stewardship process for patients with gram-negative bacteremia before and after implementation of the BioFire Blood Culture Identification 2 (BCID2) panel. The timeline depicts blood culture processing, organism identification, antimicrobial stewardship review, and antimicrobial optimization.

The conventional methodology used by microbiology included blood culture incubation using the BACTEC system (Becton, Dickinson and Company, Franklin Lakes, NJ, USA), followed by Gram staining, organism identification via matrix-assisted laser desorption/ionization–time of flight (MALDI-TOF) mass spectrometry (Bruker Daltonics, Billerica, MA, USA), and antimicrobial susceptibility testing (AST) using the MicroScan system (Beckman Coulter, Brea, CA, USA) (Fig. 1).

Clinical and microbiological data were collected from the electronic health record and laboratory information system. Variables collected included demographics, source of infection, pathogen identified, admission source, severity of illness (including ICU status, Pitt Bacteremia Score, and Charlson Comorbidity Index), antibiotic therapy details, and stewardship interventions. Time from blood culture collection to key clinical events was recorded, including times to pathogen identification, effective antimicrobial therapy, and appropriate antimicrobial therapy.

The primary outcome, time to effective antimicrobial therapy, was defined as the time from blood culture collection to administration of an empiric antimicrobial agent with presumed *in vitro* activity against the identified pathogen. Secondary outcomes included hospital length of stay, in-hospital 30-day mortality, and time to appropriate therapy, defined as the time from blood culture collection to administration of a definitive antimicrobial agent with confirmed *in vitro* activity against the identified pathogen. A subgroup analysis was completed for all extended spectrum βeta-lactamase (ESBL)-producing bacteria, which evaluated time to effective and appropriate therapy, hospital length of stay, and in-hospital 30-day mortality.

ESBL-producing organisms were identified using phenotypic susceptibility testing by MicroScan in conjunction with BCID/BCID2 results. CTX-M detection was available only in the BCID2 cohort, as this target was not included on the original BCID panel. Therefore, CTX-M carrier status could not be determined in the BCID cohort.

Based on previous studies (14, 15), a sample size of 76 patients was calculated to detect a 9-h difference in time to effective antibiotic therapy with 80% power, with a *P* value of <0.05 considered statistically significant. Continuous variables were reported as medians with interquartile ranges (IQRs) and compared using the Mann-Whitney *U* test. Categorical variables were presented as percentages and analyzed using Chi-square or Fisher’s exact tests, as appropriate. Kaplan-Meier survival curves were used to assess time to event outcomes. Exploratory linear regression analyses were performed to evaluate the association between immunocompromised status, solid organ transplant status, and time to effective antimicrobial therapy. All statistical analyses were conducted using Microsoft Excel version 16.86 and R version 4.4.2 (2024-10-31).

## RESULTS

**Fig 2:**
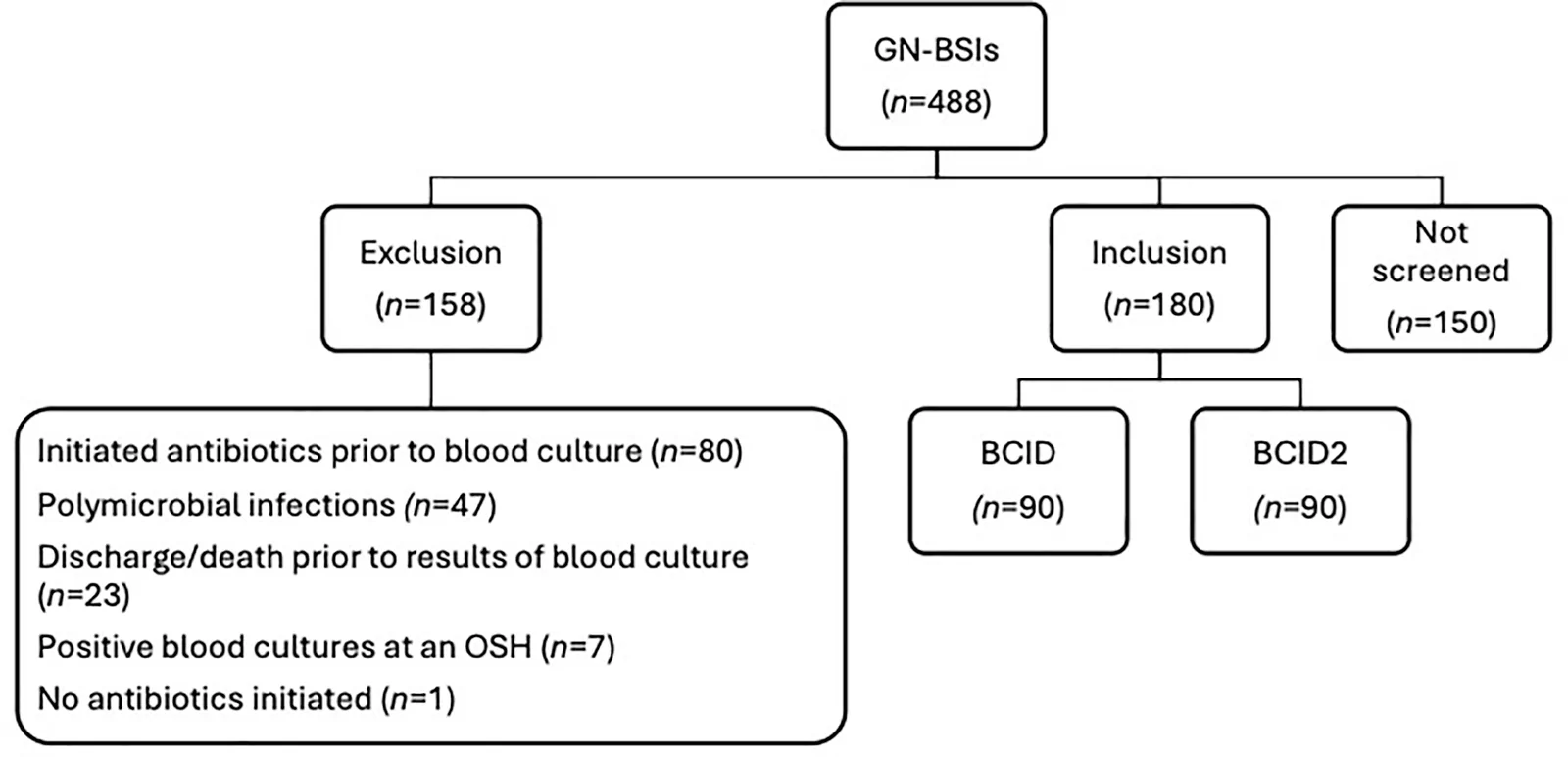
Flow diagram of study population. Flow diagram summarizing patient screening, exclusion criteria, and final cohort selection for patients included in the comparison of the BioFire Blood Culture Identification (BCID) and BioFire Blood Culture Identification 2 (BCID2) panels.

A total of 488 patients with GN-BSIs were identified during the study period. Inclusion was censored at 180 patients due to achieving power, and 158 patients were excluded based on predefined exclusion criteria (Fig. 2). A randomized selection process was used to obtain the 180 included patients. Of the 180 patients included in the study, 90 were in the pre-implementation BCID group and 90 were in the post-implementation BCID2 group. The baseline characteristics of the two groups are presented in Table 1. The median age was similar in both groups (66 years in the BCID2 group vs 69 years in the BCID group, *P* = 0.103), and most patients were admitted from home (81.1% vs 74.4%, *P* = 0.260, respectively). The median Pitt Bacteremia Score was (2 vs 2, *P* = 0.526), and the median Charlson Comorbidity Index was (4 vs 4, *P* = 0.916). Statistically significant differences were observed in the proportion of patients with a history of solid organ transplant (7.8% vs 24.4%, *P* < 0.001) and those receiving immunosuppressive therapy (17.8% vs 40.0%, *P* < 0.001), both of which were more common in the BCID group.

**TABLE 1:** Baseline characteristics

Characteristic	BCID *N* = 90	BCID2 *N* = 90	*P* value
Age (years), median (IQR)	62 (52–72)	61.5 (51–76)	0.834
Female, *n* (%)	45 (50)	40 (55.6)	0.46
Weight (kg), mean (SD)	78.5 + 20.6	78.5 + 24.6	0.726
Allergy to β-lactam, *n* (%)	14 (15.6)	7 (7.8)	0.10
Location, *n* (%)			0.87
ICU	25 (27.8)	24 (26.7)	
Non-ICU	65 (72.2)	66 (73.3)	
Admitted from, *n* (%)			
Home	69 (76.7)	68 (75.6)	
LTC^*a*^	9 (10)	7 (7.8)	
Transfer	12 (13.3)	15 (16.7)	
Hospitalization within 180 days, *n* (%)	59 (65.6)	46 (48.9)	0.07
Positive blood culture within 180 days, *n* (%)	9 (10)	11 (12.2)	0.64
Antibiotic use within 180 days, *n* (%)	39 (43.3)	32 (35.6)	0.29
History of multidrug-resistant organisms, *n* (%)	18 (20)	15 (16.7)	0.849
Charlson comorbidity index, median (IQR)	4 (2–6)	4 (2–7)	0.976
Pitt bacteremia score, median (IQR)	1 (0–2)	1 (0–2)	0.276
Solid organ transplant, *n* (%)	22 (24.4)	7 (7.8)	<0.001
Immunocompromising therapy, *n* (%)	36 (40)	16 (17.8)	<0.001
CKD, *n* (%)	20 (22.2)	16 (17.8)	0.46
DM, *n* (%)	30 (33.3)	20 (22.2)	0.10
Time to positivity, median (IQR), hours	10 (10–12.4)	11 (10–13)	0.038
Type of acquisition, *n* (%)			0.34
Hospital acquired	32 (35.6)	26 (28.9)	
Community acquired	58 (64.4)	64 (71.1)	
Source of infection, *n* (%)			
GU	41 (45.6)	42 (46.7)	
GI	23 (25.6)	35 (38.9)	
SSTI	5 (5.6)	2 (2.2)	
Pulmonary	4 (4.4)	2 (2.2)	
Catheter associated	2 (2.2)	2 (2.2)	
Endovascular	0 (0)	2 (2.2)	
Other	15 (16.6)	5 (5.5)	
Organism identified, *n* (%)			
*Escherichia coli*	51 (56.7%)	37 (41.1%)	
*Klebsiella pneumoniae*	14 (15.6%)	22 (24.4%)	
*Enterobacter cloacae* complex	9 (10%)	6 (6.7%)	
*Pseudomonas aeruginosa*	5 (5.6%)	6 (6.7%)	
*Bacteroides fragilis*	2 (2.2%)	4 (4.4%)	
*Proteus mirabilis*	3 (3.3%)	1 (1.1%)	
*Acinetobacter baumannii*	1 (1.1%)	3 (3.3%)	
*Enterobacter aerogenes*		2 (2.2%)	
*Serratia marcescens*		2 (2.2%)	
*Klebsiella oxytoca*/*Raoultella ornithinolytica*	2 (2.2%)	2 (2.2%)	
*Salmonella* species	1 (1.1%)	2 (2.2%)	
*Neisseria meningitidis*		1 (1.1%)	
*Haemophilus influenzae*	1 (1.1%)	1 (1.1%)	
*Stenotrophomonas (X.) maltophilia*	1 (1.1%)	1 (1.1%)	
ID consult, *n* (%)	71 (78.9)	61 (67.8)	0.09
ASP intervention, *n* (%)	18 (20)	18 (20)	1.0
Duration of treatment, median (IQR), days	13.5 (7–13)	10 (8–14)	0.322

^*a*^ Long-term care.

The median time to positivity was slightly longer in the BCID2 group (11 h [IQR, 10–13]) compared to the BCID group (10 h [IQR, 10–12.4], *P* = 0.038). Most of the BSIs were community-acquired (71.1% in the BCID2 group vs 64.4% in the BCID group, *P* = 0.34). The most common source of infection was genitourinary (46.7% vs 45.6%). The most frequently isolated pathogens in both groups were *Escherichia coli* (41.1% vs 56.7%), followed by *Klebsiella pneumoniae* (24.4% vs 15.6%).

In the BCID group, the most frequently used effective antibiotics were cefepime (49%), piperacillin/tazobactam (22%), and meropenem (12%), with a transition to ceftriaxone as the most common definitive therapy (26%). In comparison, the BCID2 group more commonly received cefepime (38%), piperacillin/tazobactam (30%), and ceftriaxone (20%) as initial effective therapy, with a similar transition pattern to ceftriaxone for definitive therapy (28%).

The primary outcome, time to effective antimicrobial therapy, was significantly shorter in the BCID2 group compared to the BCID group (1 h [IQR, 0–3.6] vs 4 h [IQR, 0.8–13.6], *P* = 0.004). Time to appropriate therapy was shorter, though not statistically significant, in the BCID2 group compared to the BCID group (25 h [IQR, 9.6–75.5] vs 44 h [IQR, 9.7–78], *P* = 0.627). Hospital length of stay was 10 days (IQR, 6–22) in the BCID2 group and 14 days (IQR, 7–30) in the BCID group (*P* = 0.134). In-hospital 30-day mortality was 6.7% in the BCID2 group and 8.9% in the BCID group (*P* = 0.756). All results are presented in Table 2.

**TABLE 2:** Results

Primary outcome			
Endpoint	BCID *N* = 90	BCID2 *N* = 90	*P* value
Time to effective therapy, median (IQR), hours	4 (0.8–13.6)	1 (0.3–6)	0.004

Secondary outcomes			
Time to appropriate therapy, median (IQR), hours	49 (9.7–78)	25 (9.6–75.5)	0.627
Length of stay, median (IQR), days	14 (7–30)	10 (6–22)	0.134
30-day mortality, *n* (%)	8 (8.9)	6 (6.7)	0.756

Subgroup analysis (ESBL organisms)			
Endpoint	BCID *N* = 19	BCID2 *N* = 17	*P* value
Time to effective therapy, median (IQR), hours	3 (1.2–9.5)	0.5 (0.2–2)	0.006
Time to appropriate therapy, median (IQR), hours	44 (12.9–62.3)	16 (11–17)	0.016
Length of stay, median (IQR), days	14 (7–53)	20 (9–27)	0.865
30-day mortality, *n* (%)	2 (9.5)	0 (0)	0.492

Exploratory regression analyses were performed to evaluate the association between immunocompromised status and solid organ transplant status with time to effective antimicrobial therapy. Neither immunocompromised status nor solid organ transplant status was significantly associated with the primary outcome in either the BCID cohort or the BCID2 cohort (Table S3).

A predefined subgroup analysis as presented in Table 2 was conducted in patients with ESBL-producing organisms. In this subgroup, the BCID2 group had a significantly shorter time to effective therapy compared to the BCID group (0.5 h [IQR, 0.2–2] vs 3 h [IQR, 1.2–9.5], *P* = 0.006). Time to appropriate therapy was also significantly shorter with BCID2 vs BCID (16 h [IQR, 11–77] vs 44 h [IQR, 12.9–62.3], *P* = 0.016). There were no statistically significant differences in length of stay (20 days [IQR, 9–27] vs 14 days [IQR, 7–53]; *P* = 0.865) or 30-day mortality (0% vs 9.5%; *P* = 0.492) in the ESBL subgroup analysis.

Among ESBL-producing isolates, BCID2 identified the CTX-M resistance marker in all 17 cases. Antimicrobial escalation occurred in 15 of 17 (88.2%) patients in the BCID2 cohort, most commonly from ceftriaxone, cefepime, or piperacillin/tazobactam to meropenem prior to the availability of conventional susceptibility results. In comparison, 15 of 19 (78.9%) patients in the BCID cohort underwent escalation following conventional susceptibility results. ASP intervention was documented in 7 of 17 (41.2%) BCID2 cases and 7 of 19 (36.8%) BCID cases (Tables S1 and S3).

**Fig 3:**
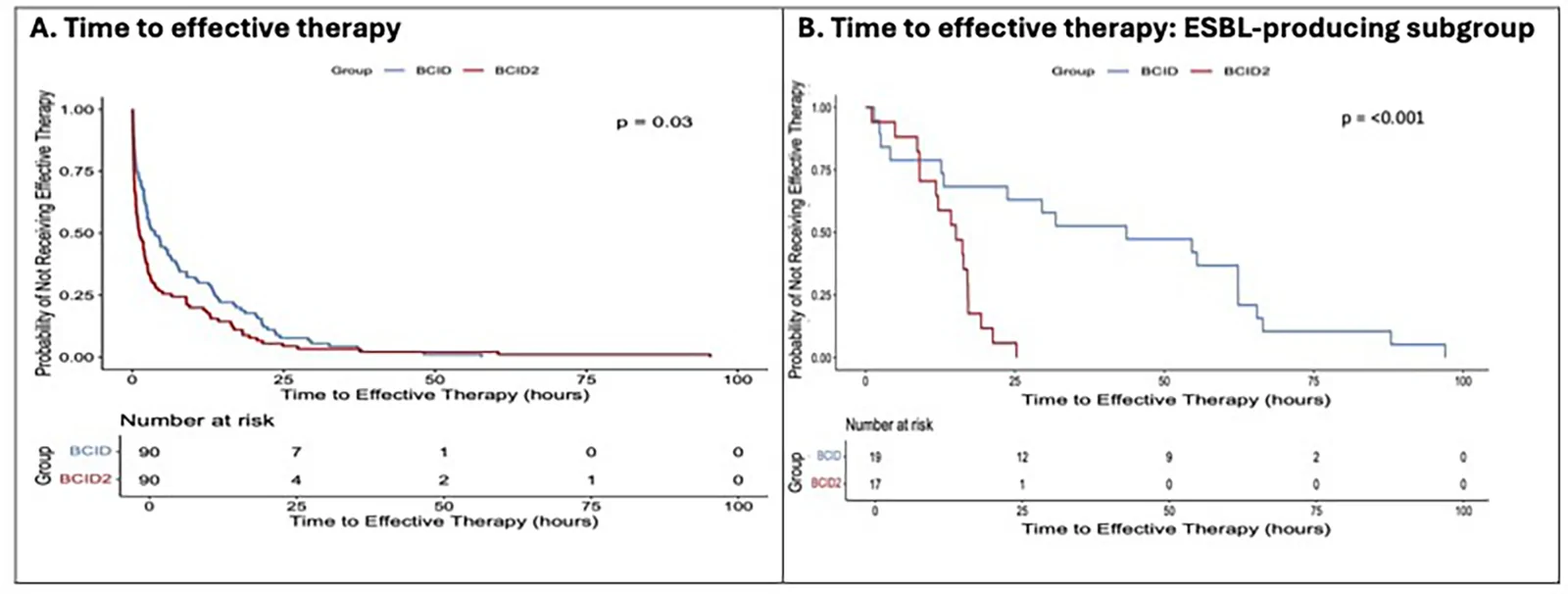
Time-to-event analysis of time to effective antimicrobial therapy. Kaplan–Meier curves comparing time to effective antimicrobial therapy between patients evaluated using the original BioFire Blood Culture Identification (BCID) panel and the BioFire Blood Culture Identification 2 (BCID2) Panel. Panel **A** depicts the overall study population, and panel **B** depicts the extended-spectrum β-lactamase (ESBL)-producing subgroup.

Kaplan-Meier survival analysis confirmed a statistically significant difference in time to effective therapy favoring BCID2 when evaluating all patients with GN-BSIs and only patients with BSIs from ESBL-producing bacteria. As shown in Fig. 3, patients in the BCID2 group had a higher probability of receiving effective therapy within 24 h in the BCID2 group in comparison to the BCID group in both the primary outcome (log-rank *P* = 0.03) and ESBL-producing subgroup (log-rank *P* < 0.001).

## DISCUSSION

In this retrospective quasi-experimental study, implementation of the BioFire BCID2 panel significantly reduced time to empiric effective antimicrobial therapy in patients with GN-BSIs particularly in the subgroup of patients with ESBL-producing organisms. This improvement is likely driven by the panel’s expanded resistance gene detection, including CTX-M. This study only focused on gram-negative BSIs because the majority of the additional resistance targets in the BCID2 panel, such as CTX-M, NDM, KPC, and OXA-48, are associated with gram-negative organisms (11). Organisms carrying these genes are frequently implicated in multidrug-resistant infections and are linked to increased morbidity and mortality, making timely identification and targeted therapy especially important (1, 3, 4, 6).

Interpretation of the primary endpoint should be considered in the context of empiric antimicrobial therapy. Across the overall study cohort, 69.4% of patients were receiving effective empiric antimicrobial therapy that either required no modification or was subsequently de-escalated, whereas 30.6% required escalation of therapy following organism identification. These findings suggest that although most patients received effective empiric therapy, rapid diagnostic testing remained clinically valuable by facilitating earlier optimization of antimicrobial therapy, particularly among patients requiring escalation.

The broader range of organisms identified in BCID2 may have contributed to earlier antimicrobial optimization in some cases. *Bacteroides fragilis*, an anaerobic pathogen associated with intra-abdominal and polymicrobial infections, is included in the BCID2 but not in the original BCID panel. These infections often require antibiotics with anaerobic coverage (i.e., such as with metronidazole, beta-lactam/beta-lactamase inhibitor combinations, and carbapenems) due to intrinsic resistance to third- and fourth-generation cephalosporins (i.e., ceftriaxone and cefepime) (11, 16, 17). In our study, two *B. fragilis* bloodstream infections were identified in the BCID group where both patients received empiric anaerobic coverage. In comparison, there were four cases of *B. fragilis* in the BCID2 group. One patient in the BCID2 group initially received ceftriaxone for a presumed gastrointestinal source, 8 hrs after blood culture collection and was then escalated to piperacillin/tazobactam with microbiological confirmation of *B. fragilis*. These findings highlight the potential added value of BCID2’s expanded organism detection in supporting earlier and more targeted treatment decisions.

Our findings are corroborated with existing literature and provide meaningful comparisons. Bae et al. demonstrated no difference in time to effective therapy, with a median of 3 h in both the BCID panel and conventional detection methods (*P* = 0.789), with ASP involvement. The ASP model employed was preauthorization and not real-time review and feedback, potentially influencing the lack of reduction in time to effective therapy (18). Similarly, Kanda et al. found no difference between BCID and BCID2 groups in simulation modeling, reinforcing the idea that the implementation of the panel significantly influences clinical outcomes (15). Our real-world study observed a statistically significant reduction in time to effective therapy with BCID2 and a contributing factor may have been our ASP structure; a pharmacist and physician-led prospective audit and feedback model that provided real-time review of positive blood cultures during business hours (Monday–Friday). These findings are consistent with other studies demonstrating the clinical benefit of rapid diagnostics in combination with ASP. Perez et al. reported that integrating rapid diagnostics with ASP significantly improved time to optimal therapy and was associated with lower mortality and shorter hospital stays in patients with antibiotic-resistant gram-negative bacteremia(19). Additionally, Henig et al. evaluated the potential benefit of the Accelerate Pheno system in 61 patients with resistant gram-negative BSIs, finding that it could have reduced the median time to effective therapy by 16.6 h and time to definitive therapy by 29.8 h (20).

Current guidelines recommend initiating empiric antimicrobial therapy as promptly as possible after obtaining blood cultures, ideally within 1 h, for patients with suspected sepsis or bloodstream infection (21, 22). While empiric therapy is commonly started within a few hours, studies show that effective therapy typically takes 1–4 h to initiate (4, 8, 23). Delays extending beyond 24 h, however, are not uncommon and are associated with higher rates of mortality (24). One study reported a median time to effective therapy of 25.9 h, a delay associated with an increased mortality risk (20). As shown in Fig. 3, the time-to-event analysis demonstrates that patients whose pathogens were identified using the BCID2 panel had a higher likelihood of receiving effective therapy within 24 h, compared to those in the BCID group, where some patients experienced delays of over 48 h before receiving effective therapy.

Although the difference was not statistically significant, our study observed a decrease in time to appropriate therapy following BCID2 implementation. Similarly, Bae et al. reported a more substantial reduction comparing conventional detection methods to the use of the BCID panel though this finding was also not statistically significant (*P* = 0.727) (18). Graff et al. found that BCID2, compared to the original BCID panel, theoretically supports a more rapid time to optimal therapy. This reduction was likewise not statistically significant (*P* = 0.3) (14). These findings corroborate with our results, suggesting that while BCID2 may contribute to reduced time to appropriate therapy, the observed reductions may not be statistically significant.

The ESBL subgroup analysis suggests a potential benefit of the BCID2 panel. In this group, both time to effective and appropriate therapy were significantly shorter in the BCID2 group, emphasizing the benefit of detecting more markers for resistance. The rapid detection of ESBL genes, such as CTX-M, facilitates earlier, more targeted therapy. This mechanism was evident in our cohort, where CTX-M was detected in all ESBL-producing isolates identified by BCID2. In most cases, clinicians escalated therapy from ceftriaxone, cefepime, or piperacillin/tazobactam to meropenem prior to the availability of conventional susceptibility testing. These findings suggest that the reduction in time to effective therapy observed in the ESBL subgroup was likely driven by earlier recognition of ESBL-mediated resistance through BCID2. Existing literature also reports improved turnaround times and appropriate therapy initiation for resistant organisms using BCID2, reinforcing our real-world outcomes (14, 15).

Both cohorts in our study demonstrated low in-hospital 30-day mortality rates. Reported 30-day mortality rates for gram-negative BSIs typically range between 15% and 25%, particularly in critically ill or immunocompromised populations (4, 5). The lower mortality observed in our study is consistent with the relatively low Pitt Bacteremia Scores and Charlson Comorbidity Indexes observed, suggesting a less severely ill cohort. Prior studies reported higher Pitt Bacteremia Scores (mean 2), possibly explaining the higher reported mortality (23). The additional exclusion of patients who died within 48 h of blood culture collection may have further influenced mortality data by omitting the most critically ill individuals. Nevertheless, ICU admission rates were similar between both groups, supporting comparability in illness severity.

Baseline differences between groups may have further influenced clinical outcomes. The BCID group included a higher proportion of immunocompromised patients, including those with a history of solid organ transplant and those receiving immunosuppressive therapy. This imbalance may have contributed to longer durations of broad-spectrum therapy and could have confounded outcomes such as length of stay and timing of appropriate therapy. Although no statistically significant difference in hospital length of stay was detected, the BCID group exhibited a numerically higher median LOS, consistent with greater immunosuppression burden. However, exploratory regression analyses did not identify a significant association between immunocompromised status or solid organ transplant status and time to effective antimicrobial therapy within either study cohort. While residual confounding remains possible given the study design, these findings suggest that differences in transplant and immunocompromised populations were unlikely to fully explain the observed reduction in time to effective therapy following BCID2 implementation.

This study has several limitations. This was a retrospective, single-center study, which may limit the generalizability of findings to other institutions with different patient populations or antimicrobial stewardship practices. Our study focused only on gram-negative bloodstream infections, excluding gram-positive and fungal infections, which may restrict broader applicability of the BCID2 panel’s full diagnostic capabilities. Additionally, the exclusion of polymicrobial infections, patients who expired or were discharged prior to organism identification, and patients who received antibiotics before blood culture collection may limit the generalizability of our findings. While these exclusions were intended to reduce confounding and allow for a more standardized assessment of antimicrobial optimization following rapid diagnostic testing, they may have excluded patients with higher acuity and more complex infections in whom rapid diagnostics could have greater clinical impact. Furthermore, the relatively low Pitt Bacteremia Scores observed in both cohorts suggest that our findings may be most applicable to a lower-acuity gram-negative bloodstream infection population. The BCID group included a higher proportion of immunocompromised patients, potentially confounding clinical outcomes such as length of stay and the timing of therapy adjustments due to differences in baseline risk. Real-time antimicrobial stewardship review of blood cultures was limited, particularly after hours and on weekends, which may have diminished the measurable impact on therapy optimization. Lastly, BCID2 panel’s expanded targets include more resistance genes and organisms, but substantial overlap with the original panel may have reduced differences in time to organism identification between groups.

### Conclusion

Implementation of the BCID2 panel in comparison to the BCID panel was associated with a significant reduction in time to effective antimicrobial therapy in patients with gram-negative bloodstream infections. These findings highlight the potential of rapid diagnostic panels with expanded resistance gene detection to enhance early therapeutic decision-making, particularly in the context of high-risk, multidrug-resistant infections. Future prospective studies are warranted to further evaluate the impact of BCID2 across a broader spectrum of bloodstream infections, including gram-positive and polymicrobial cases, and to assess its role in supporting antimicrobial stewardship and improving clinical outcomes.

## SUPPLEMENTAL MATERIAL

Tables S1 to S3 jcm.00328-26-s0001.docx Table S1: ESBL producing isolates in the BCID panel. Table S2: ESBL producing isolates in the BCID 2 panel. Table S3: Linear regression analyses.

ASM does not own the copyrights to Supplemental Material that may be linked to, or accessed through, an article. The authors have granted ASM a non-exclusive, world-wide license to publish the Supplemental Material files. Please contact the corresponding author directly for reuse.

## References

[1] Martinez RM, Wolk DM. 2016. Bloodstream infections. Microbiol Spectr 4. doi:10.1128/microbiolspec.DMIH2-0031-2016 27726765

[2] Ohnuma T, Chihara S, Costin B, Treggiari MM, Bartz RR, Raghunathan K, Krishnamoorthy V. 2023. Association of appropriate empirical antimicrobial therapy with in-hospital mortality in patients with bloodstream infections in the US. JAMA Netw Open 6:e2249353. doi:10.1001/jamanetworkopen.2022.49353 PMC9857618 36598788

[3] Peri AM, Stewart A, Hume A, Irwin A, Harris PNA. 2021. New microbiological techniques for the diagnosis of bacterial infections and sepsis in ICU including point of care. Curr Infect Dis Rep 23:12. doi:10.1007/s11908-021-00755-0 PMC8207499 34149321

[4] Timbrook TT, Morton JB, McConeghy KW, Caffrey AR, Mylonakis E, LaPlante KL. 2017. The effect of molecular rapid diagnostic testing on clinical outcomes in bloodstream infections: a systematic review and meta-analysis. Clin Infect Dis 64:15–23. doi:10.1093/cid/ciw649 27678085

[5] Van Heuverswyn J, Valik JK, Desirée van der Werff S, Hedberg P, Giske C, Nauclér P. 2023. Association between time to appropriate antimicrobial treatment and 30-day mortality in patients with bloodstream infections: a retrospective cohort study. Clinical Infectious Diseases 76:469–478. doi:10.1093/cid/ciac727 PMC9907509 36065752

[6] Kang C-I, Kim S-H, Park WB, Lee K-D, Kim H-B, Kim E-C, Oh M, Choe K-W. 2005. Bloodstream infections caused by antibiotic-resistant gram-negative bacilli: risk factors for mortality and impact of inappropriate initial antimicrobial therapy on outcome. Antimicrob Agents Chemother 49:760–766. doi:10.1128/AAC.49.2.760-766.2005 PMC547233 15673761

[7] Yang S, Rothman RE. 2004. PCR-based diagnostics for infectious diseases: uses, limitations, and future applications in acute-care settings. Lancet Infect Dis 4:337–348. doi:10.1016/S1473-3099(04)01044-8 PMC7106425 15172342

[8] Peri AM, Ling W, Furuya-Kanamori L, Harris PNA, Paterson DL. 2022. Performance of BioFire blood culture identification 2 panel (BCID2) for the detection of bloodstream pathogens and their associated resistance markers: a systematic review and meta-analysis of diagnostic test accuracy studies. BMC Infect Dis 22:794. doi:10.1186/s12879-022-07772-x PMC9585790 36266641

[9] Wolk DM, Parrott JS, Babady NE, Mochon AB, Tom R, Diel C, Dien Bard J, Harrington A, Hata DJ, Roberts AL, Boyce L, Johnson JK. 2025. The American society for microbiology’s evidence-based laboratory medicine practice guidelines for the diagnosis of bloodstream infections using rapid tests: a systematic review and meta-analysis. Clin Microbiol Rev 38:e0013724. doi:10.1128/cmr.00137-24 PMC12424361 40522178

[10] Verroken A, Despas N, Rodriguez-Villalobos H, Laterre PF. 2019. The impact of a rapid molecular identification test on positive blood cultures from critically ill with bacteremia: a pre-post intervention study. PLoS One 14:e0223122. doi:10.1371/journal.pone.0223122 PMC6762135 31557233

[11] Cortazzo V, D’Inzeo T, Giordano L, Menchinelli G, Liotti FM, Fiori B, De Maio F, Luzzaro F, Sanguinetti M, Posteraro B, Spanu T. 2021. Comparing BioFire FilmArray BCID2 and BCID panels for direct detection of bacterial pathogens and antimicrobial resistance genes from positive blood cultures. J Clin Microbiol 59:03163–20. doi:10.1128/JCM.03163-20 PMC8092734 33472903

[12] Rhoads DD, Pournaras S, Leber A, Balada-Llasat J-M, Harrington A, Sambri V, She R, Berry GJ, Daly J, Good C, Tarpatzi A, Everhart K, Henry T, McKinley K, Zannoli S, Pak P, Zhang F, Barr R, Holmberg K, Kensinger B, Lu DY. 2023. Multicenter evaluation of the BIOFIRE blood culture identification 2 panel for detection of bacteria, yeasts, and antimicrobial resistance genes in positive blood culture samples. J Clin Microbiol 61:e0189122. doi:10.1128/jcm.01891-22 PMC10281132 37227281

[13] Vyas S, Patel D, Bandali A, Giordano P, Roland R, Kessler J. 2024. Impact of BioFire blood culture identification (BCID) panels on antibiotic management of bacteremia due to select organisms. Diagn Microbiol Infect Dis 110:116384. doi:10.1016/j.diagmicrobio.2024.116384 39002446

[14] Graff KE, Palmer C, Anarestani T, Velasquez D, Hamilton S, Pretty K, Parker S, Dominguez SR. 2021. Clinical impact of the expanded biofire blood culture identification 2 panel in a U.S. children’s hospital. Microbiol Spectr 9:e0042921. doi:10.1128/spectrum.00429-21 PMC8552780 34431685

[15] Kanda N, Hashimoto H, Suzuki T, Nakamura K. 2022. Performance of the new FilmArray blood culture identification 2 panel and its potential impact on clinical use in patients with Gram-negative bacteremia. J Infect Chemother 28:1037–1040. doi:10.1016/j.jiac.2022.04.008 35428573

[16] Nagy E, Urbán E, Nord CE, ESCMID Study Group on Antimicrobial Resistance in Anaerobic Bacteria. 2011. Antimicrobial susceptibility of Bacteroides fragilis group isolates in Europe: 20 years of experience. Clin Microbiol Infect 17:371–379. doi:10.1111/j.1469-0691.2010.03256.x 20456453

[17] Brook I. 2010. The role of anaerobic bacteria in bacteremia. Anaerobe 16:183–189. doi:10.1016/j.anaerobe.2009.12.001 20025984

[18] Bae J-Y, Bae J, So M-K, Choi H-J, Lee M. 2023. The impact of the rapid blood culture identification panel on antibiotic treatment and clinical outcomes in bloodstream infections, particularly those associated with multidrug-resistant micro-organisms. Diagnostics (Basel) 13:3504. doi:10.3390/diagnostics13233504 PMC10706406 38066745

[19] Perez KK, Olsen RJ, Musick WL, Cernoch PL, Davis JR, Peterson LE, Musser JM. 2014. Integrating rapid diagnostics and antimicrobial stewardship improves outcomes in patients with antibiotic-resistant Gram-negative bacteremia. J Infect 69:216–225. doi:10.1016/j.jinf.2014.05.005 24841135

[20] Henig O, Kaye KS, Chandramohan S, Cooper CC, Lephart P, Salimnia H, Taylor M, Pogue JM. 2019. The hypothetical impact of accelerate pheno on time to effective therapy and time to definitive therapy for bloodstream infections due to drug-resistant gram-negative bacilli. Antimicrob Agents Chemother 63:e01477–18. doi:10.1128/AAC.01477-18 PMC6395913 30559141

[21] Evans L, Rhodes A, Alhazzani W, Antonelli M, Coopersmith CM, French C, Machado FR, Mcintyre L, Ostermann M, Prescott HC, et al.. 2021. Surviving sepsis campaign: international guidelines for management of sepsis and septic shock 2021. Intensive Care Med 47:1181–1247. doi:10.1007/s00134-021-06506-y PMC8486643 34599691

[22] Mermel LA, Allon M, Bouza E, Craven DE, Flynn P, O’Grady NP, Raad II, Rijnders BJA, Sherertz RJ, Warren DK. 2009. Clinical practice guidelines for the diagnosis and management of intravascular catheter-related infection: 2009 update by the infectious diseases society of America. Clin Infect Dis 49:1–45. doi:10.1086/599376 PMC4039170 19489710

[23] Banerjee R, Teng CB, Cunningham SA, Ihde SM, Steckelberg JM, Moriarty JP, Shah ND, Mandrekar JN, Patel R. 2015. Randomized trial of rapid multiplex polymerase chain reaction-based blood culture identification and susceptibility testing. Clin Infect Dis 61:1071–1080. doi:10.1093/cid/civ447 PMC4560903 26197846

[24] Lodise TP, Zhao Q, Fahrbach K, Gillard PJ, Martin A. 2018. A systematic review of the association between delayed appropriate therapy and mortality among patients hospitalized with infections due to *Klebsiella pneumoniae* or *Escherichia coli*: how long is too long? BMC Infect Dis 18. doi:10.1186/s12879-018-3524-8 PMC6280436 30518337

