# Supplementary material for: Evaluation of time to antibiotic therapy after implementation of an updated rapid diagnostic test for gram-negative bacteremia
Source: J Clin Microbiol. 2026 Aug 31;64(10):e00328-26. doi: 10.1128/jcm.00328-26 (PMC13712940; doi:10.1128/jcm.00328-26)
Supplement: Supplementary file 1 [file jcm.00328-26-s0001.docx]

**Appendix 1:**

Supplementary Table S1: ESBL Producing Isolates in the BCID Panel

| Study ID | Pathogen Identified | Susceptibility Pattern Comments | Source of Infection | Empiric Antibiotic 1 | Definitive Antibiotic 1 | Change in Antimicrobial Selection | ASP Intervention* |
| --- | --- | --- | --- | --- | --- | --- | --- |
| PT 1 | *Escherichia coli* | ESBL | Unknown | Cefepime | Meropenem | Escalation | Yes |
| PT 2 | *Klebsiella pneumoniae* | ESBL | Unknown | Cefepime | Meropenem | Escalation | No |
| PT 3 | *Escherichia coli* | ESBL | GU | Cefepime | Meropenem | Escalation | Yes |
| PT 4 | *Escherichia coli* | ESBL | GI | Zosyn | Ceftazidime-avibactam | Escalation | No |
| PT 5 | *Escherichia coli* | ESBL | Chorioamnionitis | Ampicillin | Meropenem | Escalation | No |
| PT 6 | *Escherichia coli* | ESBL | GU | Cefepime | Meropenem | Escalation | Yes |
| PT 7 | *Escherichia coli* | ESBL | GU | Ceftazidime-avibactam | Meropenem | De-escalation | Yes |
| PT 8 | *Escherichia coli* | ESBL | GU | Meropenem | Meropenem | Escalation | No |
| PT 9 | *Escherichia coli* | ESBL | GU | Meropenem | Meropenem | Escalation | No |
| PT 10 | *Escherichia coli* | ESBL | GU | Meropenem | Meropenem | Escalation | Yes |
| PT 11 | *Escherichia coli* | ESBL | GI | Zosyn | Zosyn | N/A | No |
| PT 12 | *Escherichia coli* | ESBL | GI | Meropenem | Meropenem | N/A | No |
| PT 13 | *Escherichia coli* | ESBL | GU | Cefepime | Meropenem | Escalation | No |
| PT 14 | *Escherichia coli* | ESBL | Unknown | Zosyn | Meropenem | Escalation | No |
| PT 15 | *Escherichia coli* | ESBL | GU | Zosyn | Meropenem | Escalation | Yes |
| PT 16 | *Escherichia coli* | ESBL | GI | Aztreonam | Meropenem | Escalation | No |
| PT 17 | *Escherichia coli* | ESBL | Osteomyelitis | Unasyn | Meropenem | Escalation | Yes |
| PT 18 | *Escherichia coli* | ESBL | GU | meropenem | Meropenem | N/A | No |
| PT 19 | *Escherichia coli* | ESBL | GU | Cefepime | Meropenem | Escalation | No |

ESBL, extended-spectrum beta-lactamase; GU, genitourinary; GI, gastrointestinal, *Does not include recommendation made by primary ID consultant teams only ASP teams

Supplementary Table S2: ESBL Producing Isolates in the BCID 2 Panel

| Study ID | Pathogen Identified | Resistance Marker Detected | Source of Infection | Empiric Antibiotic 1 | Definitive Antibiotic 1 | Change in Antimicrobial Selection | ASP Intervention* |
| --- | --- | --- | --- | --- | --- | --- | --- |
| PT 1 | *Escherichia coli* | CTX-M | GU | Meropenem | Meropenem | N/A | No |
| PT 2 | *Klebsiella pneumoniae* | CTX-M | GI | Ceftriaxone | Meropenem | Escalation | No |
| PT 3 | *Klebsiella pneumoniae group* | CTX-M | GU | Cefepime | Meropenem | Escalation | Yes |
| PT 4 | *Escherichia coli* | CTX-M | GI | Zosyn | Meropenem | Escalation | Yes |
| PT 5 | *Escherichia coli* | CTX-M | GU | Ceftriaxone | Meropenem | Escalation | No |
| PT 6 | *Escherichia coli* | CTX-M | GU | Zosyn | Meropenem | Escalation | Yes |
| PT 7 | *Klebsiella pneumoniae* | CTX-M | GU | Zosyn | Meropenem | Escalation | No |
| PT 8 | *Escherichia coli* | CTX-M | GU | Cefepime | Meropenem | Escalation | No |
| PT 9 | *Escherichia coli* | CTX-M | GU | Cefepime | Meropenem | Escalation | No |
| PT 10 | *Escherichia coli* | CTX-M | GU | Cefepime | Meropenem | Escalation | No |
| PT 11 | *Klebsiella pneumoniae* | CTX-M | GU | Cefepime | Meropenem | Escalation | Yes |
| PT 12 | *Escherichia coli* | CTX-M | GI | Cefepime | Meropenem | Escalation | No |
| PT 13 | *Escherichia coli* | CTX-M | GU | Ceftriaxone | Meropenem | Escalation | Yes |
| PT 14 | *Klebsiella pneumoniae* | CTX-M | GU | Zosyn | Meropenem | Escalation | No |
| PT 15 | *Escherichia coli* | CTX-M | GI | Cefepime | Meropenem | Escalation | No |
| PT 16 | *Escherichia coli* | CTX-M | GU | Zosyn | Meropenem | Escalation | Yes |
| PT 17 | *Enterobacter cloacae complex* | CTX-M | GU | Zosyn | Zosyn | N/A | Yes |

CTX-M, cefotaximase-Munich; GU, genitourinary; GI, gastrointestinal, *Does not include recommendation made by primary ID consultant teams only ASP teams

Supplementary Table S3: Multivariable linear regression analysis of factors associated with time to effective and appropriate antimicrobial therapy.

| **Cohort** | **Outcome** | **Variable** | **β (95% CI)** | **p-value** | **R²** | **Adj R²** |
| --- | --- | --- | --- | --- | --- | --- |
| BCID | TTEA | Immunocompromised | 1.06 (-5.84 to 7.95) | 0.761 | 0.020 | -0.002 |
| BCID | TTEA | SOT | 2.87 (-4.99 to 10.73) | 0.470 | 0.020 | -0.002 |
| BCID | TTAT | Immunocompromised | -18.30 (-54.90 to 18.30) | 0.323 | 0.012 | -0.011 |
| BCID | TTAT | SOT | 11.88 (-29.84 to 53.61) | 0.573 | 0.012 | -0.011 |
| BCID2 | TTEA | Immunocompromised | -1.85 (-11.35 to 7.65) | 0.700 | 0.003 | -0.020 |
| BCID2 | TTEA | SOT | -0.02 (-13.58 to 13.54) | 0.998 | 0.003 | -0.020 |
| BCID2 | TTAT | Immunocompromised | -1.85 (-11.35 to 7.65) | 0.700 | 0.003 | -0.020 |
| BCID2 | TTAT | SOT | -0.02 (-13.58 to 13.54) | 0.998 | 0.003 | -0.020 |

*Data are presented as beta coefficients (β) with 95% confidence intervals (CI) and p-values. Models were adjusted for immunocompromised status and solid organ transplant (SOT). Outcomes are reported in hours. Abbreviations: TTEA, time to effective antimicrobial therapy; TTAT, time to appropriate therapy; SOT, solid organ transplant. BCID cohort (n=90); BCID2 cohort (n=90). Model p-values were not statistically significant for any analysis.*
